# Eggsplorer: a rapid plant–insect resistance determination tool using an automated whitefly egg quantification algorithm

**DOI:** 10.1186/s13007-023-01027-9

**Published:** 2023-05-20

**Authors:** Micha Gracianna Devi, Dan Jeric Arcega Rustia, Lize Braat, Kas Swinkels, Federico Fornaguera Espinosa, Bart M. van Marrewijk, Jochen Hemming, Lotte Caarls

**Affiliations:** 1 Plant Breeding, Wageningen University & Research, Po Box 384, 6700 AJ Wageningen, The Netherlands; 2 Greenhouse Horticulture and Flower Bulbs, Wageningen Plant Research, Wageningen University & Research, 6708 PB Wageningen, The Netherlands

**Keywords:** Insect egg quantification, Rapid phenotyping, Whitefly, Deep learning, Plant insect resistance, Bioassay

## Abstract

**Background:**

A well-known method for evaluating plant resistance to insects is by measuring insect reproduction or oviposition. Whiteflies are vectors of economically important viral diseases and are, therefore, widely studied. In a common experiment, whiteflies are placed on plants using clip-on-cages, where they can lay hundreds of eggs on susceptible plants in a few days. When quantifying whitefly eggs, most researchers perform manual eye measurements using a stereomicroscope. Compared to other insect eggs, whitefly eggs are many and very tiny, usually 0.2 mm in length and 0.08 mm in width; therefore, this process takes a lot of time and effort with and without prior expert knowledge. Plant insect resistance experiments require multiple replicates from different plant accessions; therefore, an automated and rapid method for quantifying insect eggs can save time and human resources.

**Results:**

In this work, a novel automated tool for fast quantification of whitefly eggs is presented to accelerate the determination of plant insect resistance and susceptibility. Leaf images with whitefly eggs were collected from a commercial microscope and a custom-built imaging system. A deep learning-based object detection model was trained using the collected images. The model was incorporated into an automated whitefly egg quantification algorithm, deployed in a web-based application called Eggsplorer. Upon evaluation on a testing dataset, the algorithm was able to achieve a counting accuracy as high as 0.94, *r*^2^ of 0.99, and a counting error of ± 3 eggs relative to the actual number of eggs counted by eye. The automatically collected counting results were used to determine the resistance and susceptibility of several plant accessions and were found to yield significantly comparable results as when using the manually collected counts for analysis.

**Conclusion:**

This is the first work that presents a comprehensive step-by-step method for fast determination of plant insect resistance and susceptibility with the assistance of an automated quantification tool.

## Background

Whiteflies are insects classified in the Aleyrodidae family and consist of more than 1500 species [21]. Their presence is sufficient to cause serious crop yield loss, e.g., damage by *Bemisia tabaci* (Gennadius, 1889), a very invasive whitefly species. It takes approximately 20 days during warm weather conditions for a whitefly to develop from an egg to a crawler, through to pupae, and finally an adult. Female whiteflies originate from fertilized eggs, while males originate from unfertilized eggs; the typical sex ratio is 2:1, females to males [33]. Whiteflies are phloem feeders, which means they use their four interlocked stylets to enclose food and a salivary canal allowing independent movements between plant mesophyll cells [27]. While feeding, they secrete saliva as a lubricant during the penetration of the stylets, which contain enzymes and metabolites, thereby providing protection against plant wound response [13]. Furthermore, whiteflies indirectly cause damage to plants by acting as a vector of viruses, including more than 100 plant viruses, such as those in the *Begomovirus* genus, which causes tomato yellow leaf curl virus, and viruses in the *Crinivirus* genus, which causes tomato chlorosis virus [17]. Whiteflies are predominantly polyphagous, e.g., *B. tabaci* has a wide host range such as tomato, cucumber, cotton, and sweet potato [30]. The two most dominant *B. tabaci* biotypes are Middle East-Minor Asia 1 (MEAM1 or biotype B) and Mediterranean (MED or biotype Q) genetic groups. These two biotypes have caused serious yield losses in more than 60 countries [23] with annual losses of more than one billion dollars [15].

Research works on finding whitefly resistance, especially involving wild Solanaceae accessions, have yielded Quantitative Trait Loci (QTL) for adult survival and oviposition [2, 10, 20, 29, 34, 36]. The most common methods employed to test whitefly host compatibility in greenhouse and laboratory settings are choice and no-choice assays [35]. Choice assays allow whiteflies to choose between several plant genotypes to assess whitefly preference, while no-choice assays limit whiteflies to feed on a single plant to evaluate response. A choice assay can be prepared using a Y-tube olfactometer setup for whole plants or leaves, which evaluates relative preference based on volatile compounds as well as other phenotypic characteristics such as insect mortality and oviposition [16]. On the other hand, a no-choice assay can be performed by using clip-on cages on leaves [3].

Two of the most important observation parameters obtained from both choice and no-choice assays are adult insect survival and oviposition rate. Oftentimes, the quantification of adult survival and oviposition are manually done by looking through a stereomicroscope [38]. Counting adult survival in a clip-cage setting is quick but counting the number of eggs deposited by the insects on each leaf is very laborious. Each whitefly egg is approximately 0.2 mm in length and 0.08 mm in width while its color ranges from translucent green to brown, depending on maturity. On a susceptible potato line, 5 female whiteflies, inside a 2 cm diameter clip-cage, can lay more than 100 eggs in 5 days [25]. In a natural setting, whiteflies deposit eggs at the abaxial of the leaves, thereby hiding each egg from predators and environmental factors such as precipitation and high-intensity ultraviolet light [26]. Moreover, some leaves have thick veins, trichomes, and surface unevenness that may conceal eggs and hinder observation. Furthermore, whitefly eggs are commonly found in leaf regions surrounded by whitefly honeydew, which hinders observation [37]. Whitefly honeydew provides optimal conditions for mold to grow and as protection for the development of whitefly eggs to instars [8]. Due to the aforementioned factors, researchers would benefit from a more reliable and systematic protocol for analyzing whitefly assay samples.

To assure the reliability of performed assays, microscopic images are stored for further analysis. One of the ways to analyze microscopic images is by digital image processing. Digital image processing is a computerized method for automatically identifying and detecting characteristics of objects in an image by performing operations such as color conversion, edge detection, color segmentation, and blob analysis. Digital image processing has been employed in microscopic image analysis such as for mosquito egg counting [11, 19], and beetle egg counting [12]. In the works mentioned, it involves a user that specifies algorithm parameters to optimize the image analysis results; therefore, considering it as a semi-automated approach. Unlike digital image processing-based algorithms, a deep learning-based algorithm is more resistant to variations in image appearance and requires less input from a user. Deep learning is a subset of artificial intelligence that aims to train a neural network model to learn feature hierarchies from a given dataset. Deep learning models are capable of accurately detecting minute objects, such as insect eggs, even without manually tuning algorithm parameters. Deep learning has been used in microscopic image analysis for counting nematodes [1, 18], stomata [9], and protozoan parasites [39].

This work aims to develop a novel and more efficient protocol for automated whitefly egg quantification to accelerate the determination of insect-resistant and susceptible plant accessions. The specific objectives are: (1) the development of a fast and accurate whitefly egg quantification algorithm; (2) designing a web-based platform to deploy the algorithm; (3) assessing the advantages and disadvantages of using different imaging setups for collecting leaf image assays; and (4) determining plant–insect resistance and susceptibility based on the automatically obtained egg counts. This work proves the benefits of employing novel computer techniques to achieve more objective research results in the field of plant–insect resistance.

## Materials and method

### ***Plant materials and growth conditions***

Wild (*Solanum berthaultii* (Hawkes, 1963)—BER481-3) and cultivated (*S. tuberosum* cv. RH89-039-16) potatoes were obtained from the Wageningen University and Research (WUR) Plant Breeding collection, Wageningen, The Netherlands. For collecting leaf images, in vitro propagated cuttings were grown for 2 weeks in MS20 medium, and successively transferred to the greenhouse in ⌀14 cm pots with potted soil for 3 weeks before the whitefly infection assays. Resistant wild tomato plants (*S. habrochaites* (Knapp & Spooner, 1999)—LA1777) and susceptible cultivated tomato plants (*S. lycopersicum* cv. Moneymaker) were also used in this study. Seeds were obtained from WUR Plant Breeding Department, Wageningen, The Netherlands. Seeds were sown on germination media for 2 weeks before being transplanted into ⌀14 cm pots with potting soil for 3 weeks before the whitefly infestation assays were commenced. The plants were grown in peat soil in an insect-proof greenhouse at Unifarm with a 16 h light and 8 h dark photoperiod, 21 °C/19 °C (day/night) and 70% relative humidity from September–October 2022 in Wageningen, The Netherlands.

### ***Whitefly assay***

Whitefly assays were conducted on 5-week-old plants; 3 plants per genotype. Non-viruliferous whiteflies (*B. tabaci* group Mediterranean-Middle East-Asia Minor I), reared on *S. lycopersicum* cv. Forticia from the WUR Plant Breeding Department were used for screening. No-choice assays were carried out in an insect greenhouse of WUR. The assay was done by attaching two clip-on-cages ⌀2 cm containing five synchronized 1-day-old female *B. tabaci* whiteflies on the abaxial side of the second and third fully expanded leaf of each plant. Five days later, the leaves attached with clip-cages were harvested for image acquisition, egg quantification (OR) and adult survival (AS) on the same day. Leaves can optionally be stored on wet filter paper and in 4 °C to maintain cell turgidity and prevent dehydration. AS and OR for tomato plants [20] were calculated according to the following equations, respectively:

1 $$ {\text{AS}} = \left( {\frac{alive\;whiteflies}{{total\;whiteflies}}} \right)\;survival \;for\;5\;days, $$

2 $$ {\text{OR}} = \frac{2 \times number\;of\;eggs}{{alive\;whiteflies + total\;whiteflies}}eggs\;female^{ - 1} \;for\;5\;days. $$

Arcsine transformation was used to normalize AS, whereas square root transformation was used for oviposition rate. Statistical analyses via t-tests were performed using Python 3.8.13, with the support of SciPy scientific computing library v1.9.3.

### Leaf image acquisition

**Fig. 1:**
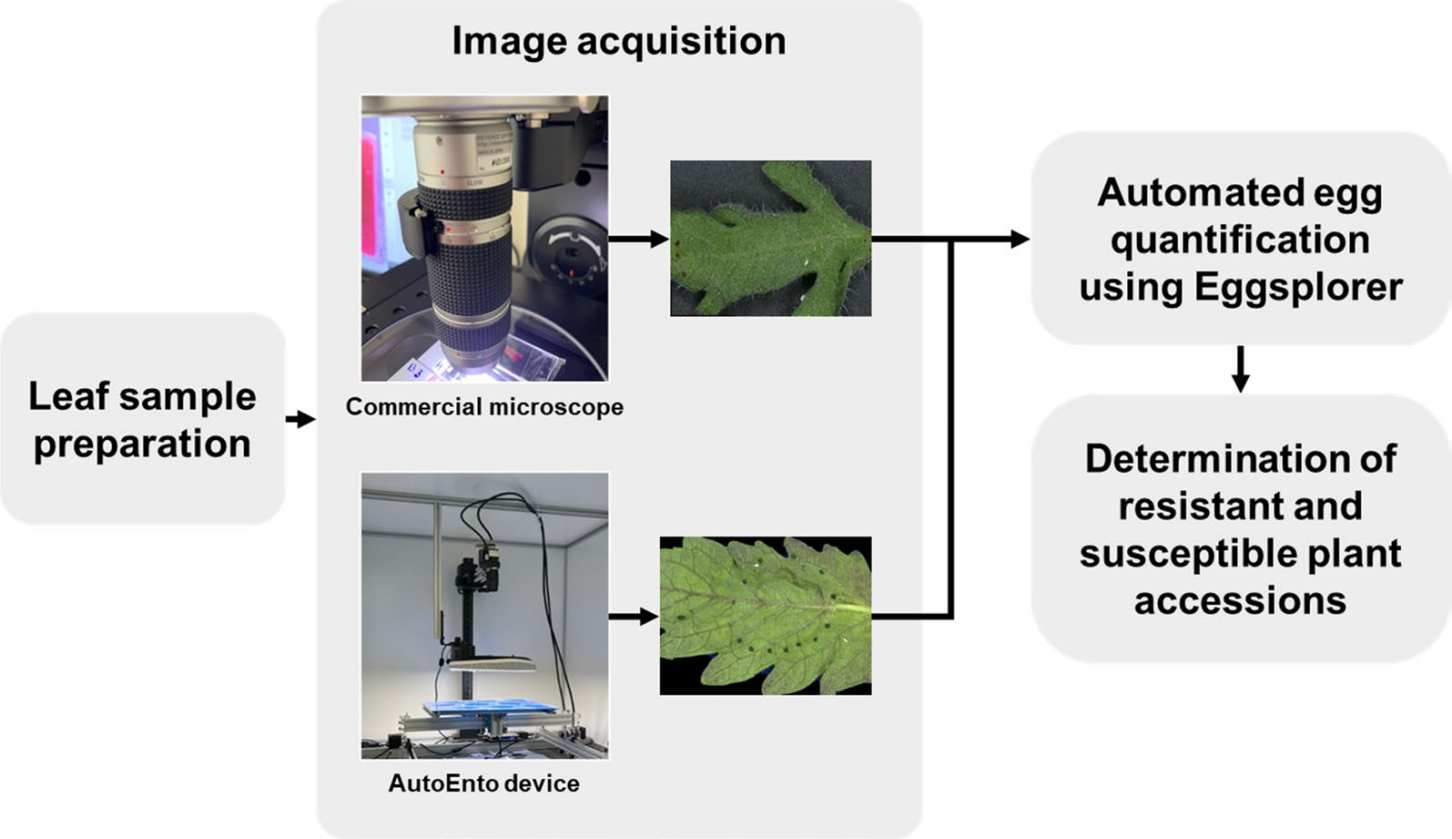
Workflow for rapid determination of plant resistance based on insect oviposition

The leaf image samples used in the whitefly assay were acquired using two different imaging setups: (1) using a commercial microscope (VHX-7000) (Keyence, Japan), and 2) using AutoEnto device, as shown in Fig. 1. The VHX-7000 is a 4K digital microscope designed for surface microscopy. It acquires high resolution leaf images by acquiring tiled images based on box vertices that were manually selected using its accompanying controller. The tiled images were automatically stitched together by its software. The VHX-7000 was electronically adjusted to set a 45–50 mm distance from the lens to the camera while the magnification was set to 30×. The automatic white balance and brightness were manually adjusted and kept uniform throughout the trials.

Meanwhile, the AutoEnto device is an imaging system developed by the Greenhouse Horticulture & Flower bulbs Business Unit of Wageningen Plant Research in Bleiswijk, The Netherlands. It is designed to rapidly acquire images of tiny biological samples such as insects, insect eggs, nematodes, and alike. It is equipped with an industrial CMOS color camera (IDS Imaging Development Systems GmbH, Germany), replaceable C-mount lenses, and a custom x–y table that can hold up to 9 dishes, for automated image acquisition and analysis. In this work, it was configured with a 30× magnification C-mount lens (Kowa Company, Japan), acquiring 4912 × 3684 pixels per image with a spatial resolution of 350 pixel/mm. In this research, it was configured to take 35 images with 4912 × 3684 pixels over a 5 × 7 grid that were stitched together into a single image. The AutoEnto device was only used for image acquisition but not for image analysis.

### Automatic egg quantification algorithm

**Fig. 2:**
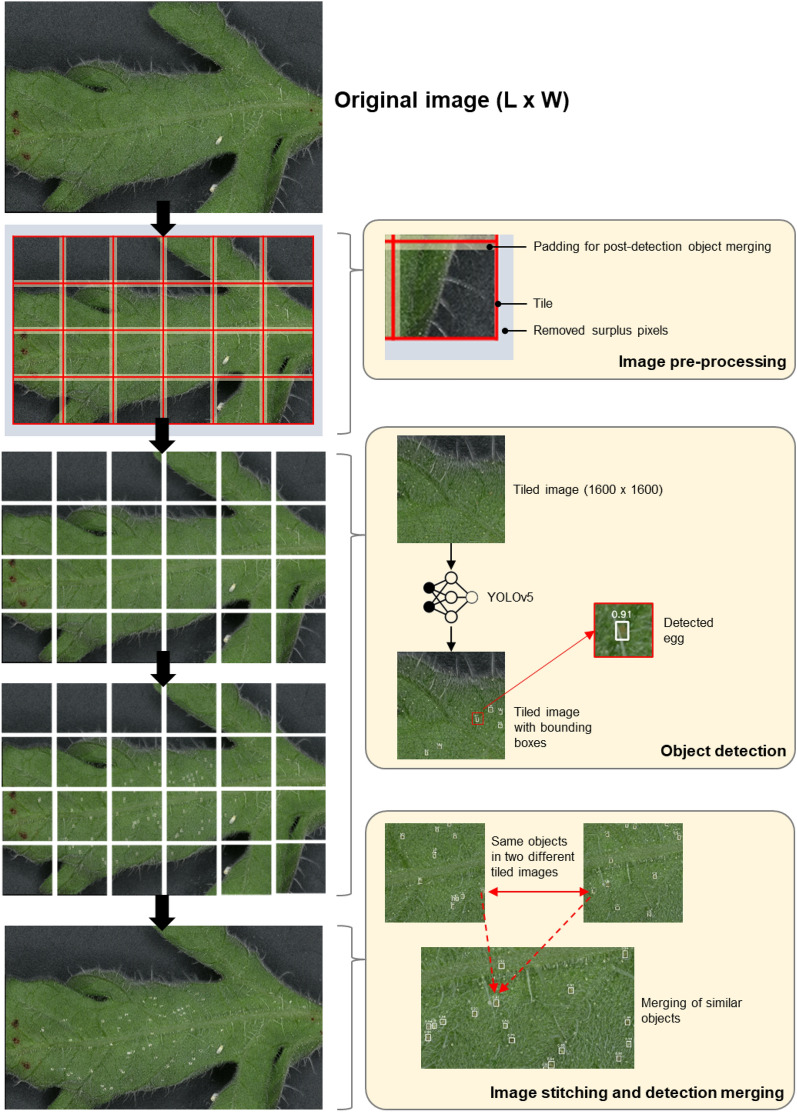
Automated egg quantification algorithm

The acquired leaf images were analyzed using an automatic egg quantification algorithm, as illustrated in Fig. 2. The egg quantification algorithm was developed using Python 3.8.3 programming language, with the support of OpenCV image processing library [5], PyTorch deep learning library [24] and MLFlow machine learning tracking library [6]. All computations were performed using a desktop computer running under Ubuntu 22.04 operating system, with an Intel Xeon E5-1650 processor, NVIDIA GeForce GTX Titan X GPU, and 16 GB RAM. This section discusses the methods and theoretical considerations in developing the algorithm.

#### Dataset preparation and image pre-processing

A leaf image dataset was prepared using the image pre-processing step of the algorithm. First, the size of the leaf image is reduced from *L* × *W* to *L* − *S*_*L*_ × *W* − *S*_*W*_ based on a pre-defined tiling size, where *L* and *W* are the length and width of the leaf image while *S*_*L*_ and *S*_*W*_ are the surplus length pixels and surplus width pixels, respectively; this was done by equally removing the pixels from all sides of the leaf image. Removal of the surplus pixels was done to attain an equal tiling size. In this work, a tiling size of 1400 × 1400 pixels was used on images obtained from both setups, with a padding of 100 pixels on all sides of the tiled leaf image. Tiling is a technique in deep learning which cuts an image into several equal parts to achieve better object detection results [28]. On the other hand, the padding allows the merging of duplicate egg detections after obtaining the detection results from each tiled leaf image later. The tiling process produces *n* 1600 × 1600 tiled leaf images that are used as individual inputs to the deep learning-based object detection model. Every egg on each tiled leaf image was annotated using a rectangular box via Darwin v7 image annotation platform with the assistance of experts. The statistical summary of the prepared image dataset is shown in Table 1.

**Table 1:** Image dataset statistical information

Device	Dataset	Size (pixels)	Total	Training	Validation	Testing
Commercial microscope (VHX-7000)	Complete image	Min.: 5258 × 6660 Ave.: 7556 × 8188 Max.: 9287 × 10,399	144	–	–	30
	Tiled image	1600 × 1600	765	35% of total	15% of total	–
	Whitefly egg	Min.: 27 × 29 Ave.: 45 × 51 Max.: 69 × 70	–	–	–	–
AutoEnto	Complete image	24,560 × 25,788	22	–	–	30
	Tiled image	1600 × 1600	413	35% of total	15% of total	
	Whitefly egg	Min.: 22 × 26 Ave.: 40 × 44 Max.: 78 × 88	–	–	–	–

#### Object detection and image stitching

The automated egg quantification algorithm uses a YOLOv5m (Ultralytics, Los Angeles, CA, USA) deep learning model for detecting the eggs from each tiled leaf image. YOLOv5m is an object detection model with three components: backbone, neck, and head. It uses cross-stage partial networks as the backbone for feature extraction while the neck, made of path aggregation networks, combines the extracted features. The combined features are used as input to a YOLO layer, which acts as the model’s head, for obtaining bounding box predictions. In this work, the YOLOv5m was specifically chosen since it has a good balance of speed and performance compared to the other YOLOv5 variants. The YOLOv5m model’s input was set to the default 614 × 614 pixels, thereby resizing each 1600 × 1600 tiled image to 614 × 614 pixels for inference. The YOLOv5m model’s output was defined to have a single image class, *egg* class. After detecting the eggs from each tiled leaf image, the leaf image was stitched back together while retranslating the bounding box coordinates according to the original leaf image size.

#### Detection post-processing

Three detection post-processing methods were applied to reduce the algorithm errors: detection merging, object size filtering, and confidence thresholding. In detection merging, similar objects found in adjacent tiled images were merged using Intersection-over-Union (*IoU*). *IoU* is a measure of the overlap between two objects in an image. *IoU* values closer to 1 indicate higher overlap and 0 otherwise. *IoU* was computed using Eq. 3:

3 $$ IoU = \frac{{area\left( {B_{1} \cap B_{2} \cap \ldots B_{o} } \right)}}{{area\left( {B_{1} \cup B_{2} \cup \ldots B_{o} } \right)}}, $$

where *B*_*o*_ is the bounding box coordinates of each detected object, with *o* as the object index. *B*_*o*_ includes four coordinates: *x*_1_, *y*_1_, *x*_2_, and *y*_2_, where *x*_1_ and *y*_1_ belong to the object’s *x* and *y* vertex box coordinates, and *x*_2_ and *y*_2_ belong to the vertex opposite to *x*_1_ and *y*_1_. If the *IoU* of two or more egg detection boxes is greater than 0.5, the boxes are merged by retaining the lowest *x*_1_ and *y*_1_ and highest *x*_2_ and *y*_2_ and counting the overlapping objects as a single object.

The object size filtering threshold was manually determined using the average size of each detected object based on Table 1. If the length or width of a detected object is less than 20 pixels, then the detected object was ignored. Such detected objects are trichomes and leaf spots that resemble the appearance of whitefly eggs. If the length or width of a detected object is more than 90 pixels, then the detected object was also ignored since such objects may include nymphs or other unwanted objects.

Confidence thresholding utilizes the confidence score of each detection, which ranges from 0 to 1.0, where values closer to 1.0 indicate higher confidence. To ignore detected objects with low confidence scores, a pre-set classification confidence threshold was defined and fine-tuned. Some of the detected objects with low confidence scores include trichomes and leaf spots.

### ***Algorithm evaluation***

The algorithm was evaluated using two methods: object-level testing and image level testing, with the test dataset defined in Table 1. In this context, object-level testing refers to how the model performs when tested on each object of all the leaf images. On the other hand, image-level testing refers to the overall algorithm performance when tested on individual leaf images. In object-level testing, the algorithm performance was evaluated by automatically matching each set of ground truth box coordinates with each predicted box coordinates via *IoU*. If the calculated *IoU* between two paired coordinates was higher than 0.5, it was counted as a true positive (TP) detection. All unmatched ground truth box coordinates were considered as missed detections. Using the above definitions, the following metrics were calculated:

4 $$ Precision = \frac{TP }{{total\;number\;of\;detected\;egg\;objects}}, $$

5 $$ Recall = \frac{TP }{{true\;number\;of\;egg\;objects}}, $$

6 $$ F_{1} score = 2 \cdot \frac{precision \cdot recall}{{precision + recall}}. $$

*F*_1_-score is a performance metric that balances precision and recall; it ranges from 0 to 1, where values closer to 1 indicate better performance. Meanwhile, the miss rate measures how many detections were undetected by the algorithm; it ranges from 0 to 1, where values closer to 1 indicate worse performance.

In image-level testing, counting accuracy, miss rate, and coefficient of determination (*r*^*2*^) were measured. Counting accuracy was measured by obtaining the percent difference between the true counts (TC) and the automatic counts (AC) per leaf image, as follows:

7 $$ Counting\;accuracy = \frac{TC - AC}{{TC}}. $$

Meanwhile, the miss rate is the ratio of missed detections and the true number of egg objects in a leaf image, computed as follows:

8 $$ Miss\;rate = \frac{missed\;detections\;in\;a\;leaf\;image}{{true\;number\;of\;egg\;objects\;in\;a\;leaf\;image}}. $$

Finally, *r*^*2*^ measures the performance of the model relative to the manual counts.

## Results and discussion

### Model training and algorithm optimization results

**Fig. 3:**
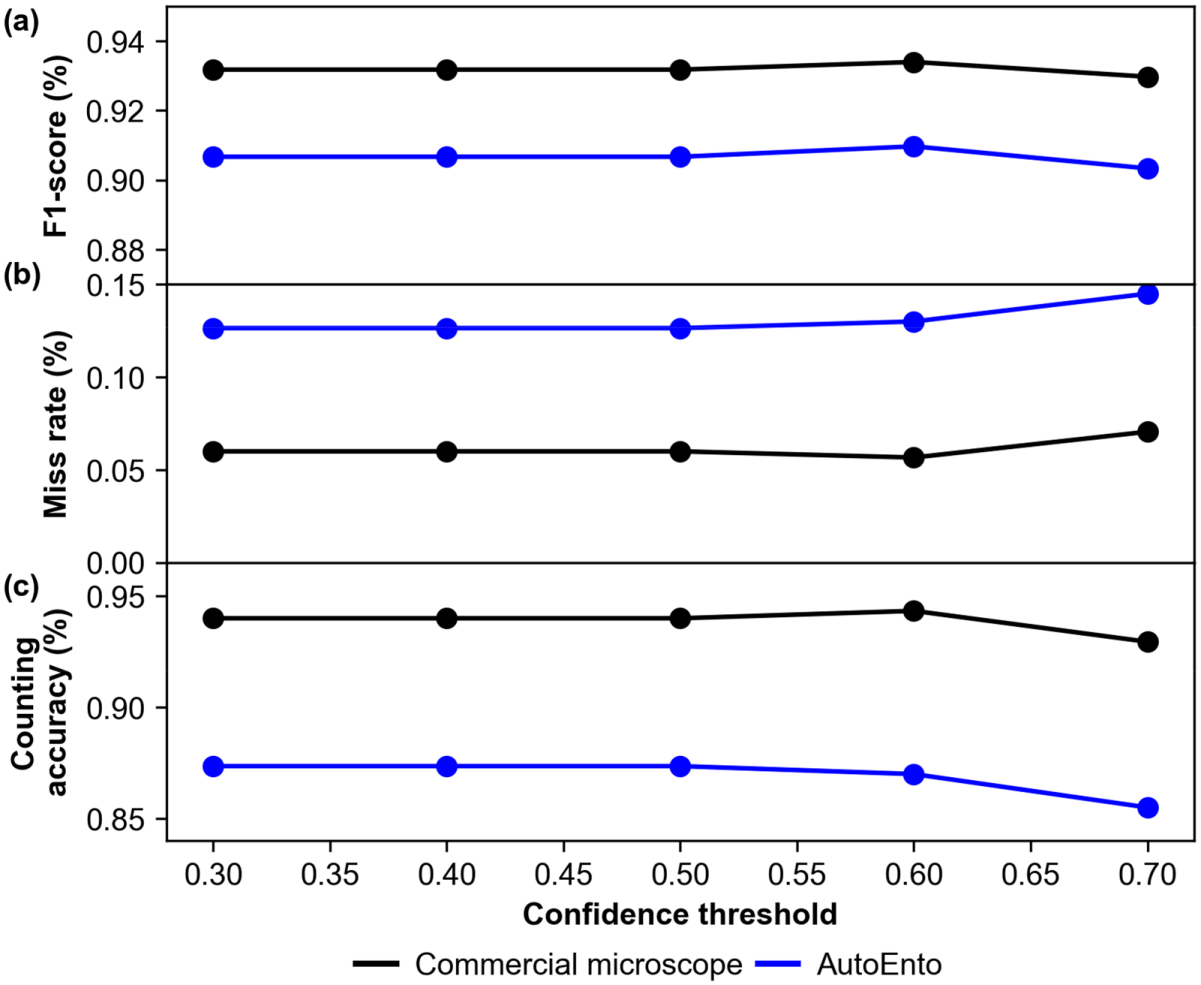
Automated egg quantification algorithm confidence threshold optimization results

In order to optimize the process of training the YOLOv5 model, hyperparameter tuning using genetic algorithm was applied [14]. Hyperparameter tuning aims to maximize model performance by finding the best training parameters such as for learning rate, momentum, box loss gain, and more. In so doing, the default YOLOv5m training hyperparameters were changed including learning rate from 0.003 to 0.01, momentum from 0.8 to 0.93, and box loss gain from 0.03 to 0.06. Training was done for 100 epochs and a batch size of 16, achieving a mean average precision of 0.96. Threshold optimization was done by fine-tuning the values of the confidence threshold from 0.3 to 0.7, with increments of 0.05. The results of threshold optimization for the two imaging setups are shown in Fig. 3.

In object level testing, the *F*_1_-score of the model can reach as high as 0.935 on images obtained using the commercial microscope and 0.91 on images obtained using the AutoEnto when using a confidence threshold of 0.6 (Fig. 3a). A slight difference in performance was expected since the commercial microscope can acquire sharper images than the AutoEnto. Nevertheless, both results show that the model detected the whitefly eggs with high accuracy and confidence. In image level testing, it was found that miss rates of the algorithm were 0.06 and 0.14 when processing the leaf images obtained by the commercial microscope and the AutoEnto, respectively, with a confidence threshold of 0.6 (Fig. 3b). The miss rate was higher using the AutoEnto since there were some parts of the leaf images that were blurred due to curling, while the commercial microscope can resolve this problem through its depth correction feature. Finally, the counting accuracies of the algorithm was about 0.94 when processing the commercial microscope leaf images and using a confidence threshold of 0.6 but had a lower counting accuracy of 0.88 when processing the AutoEnto leaf images (Fig. 3c). Based on the tuning results, an optimal confidence threshold of 0.6 was found and used throughout this research. It can be concluded that the trained model was reliable for both imaging setups, but improvements can still be made to enhance algorithm performance on images acquired using the AutoEnto.

### Algorithm testing

**Fig. 4:**
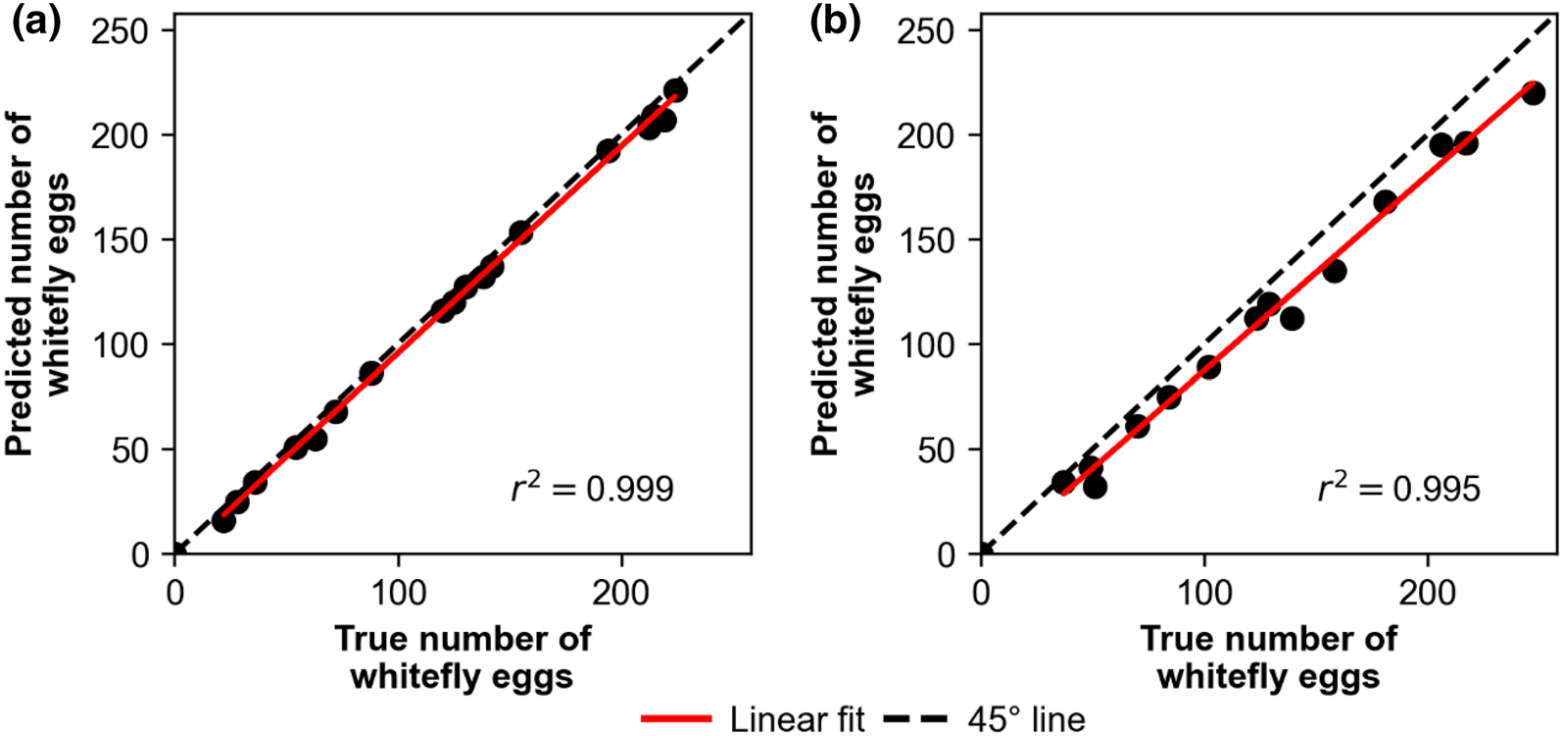
Predicted number of whitefly eggs vs. true number of whitefly eggs *r*^2^ scatter plots obtained using different imaging setups: **a** Commercial microscope; and **b** AutoEnto

The true number of eggs and predicted number of eggs were compared as shown in Fig. 4. It can be immediately seen that the predicted number of eggs from the commercial microscope images were remarkably close to the true number of eggs, with an error of ± 3 eggs relative to the actual number of eggs counted by eye, even for high number of whitefly eggs (> 200 eggs). On the other hand, the algorithm still performed well with minor issues when analyzing the AutoEnto leaf images, with an error of ± 10 eggs. As mentioned previously, this was mainly caused by blurred spots which cause missed whitefly egg detections. In summary, this shows that the algorithm is usable in both imaging setups and can accurately estimate the number of whitefly eggs in each leaf.

**Fig. 5:**
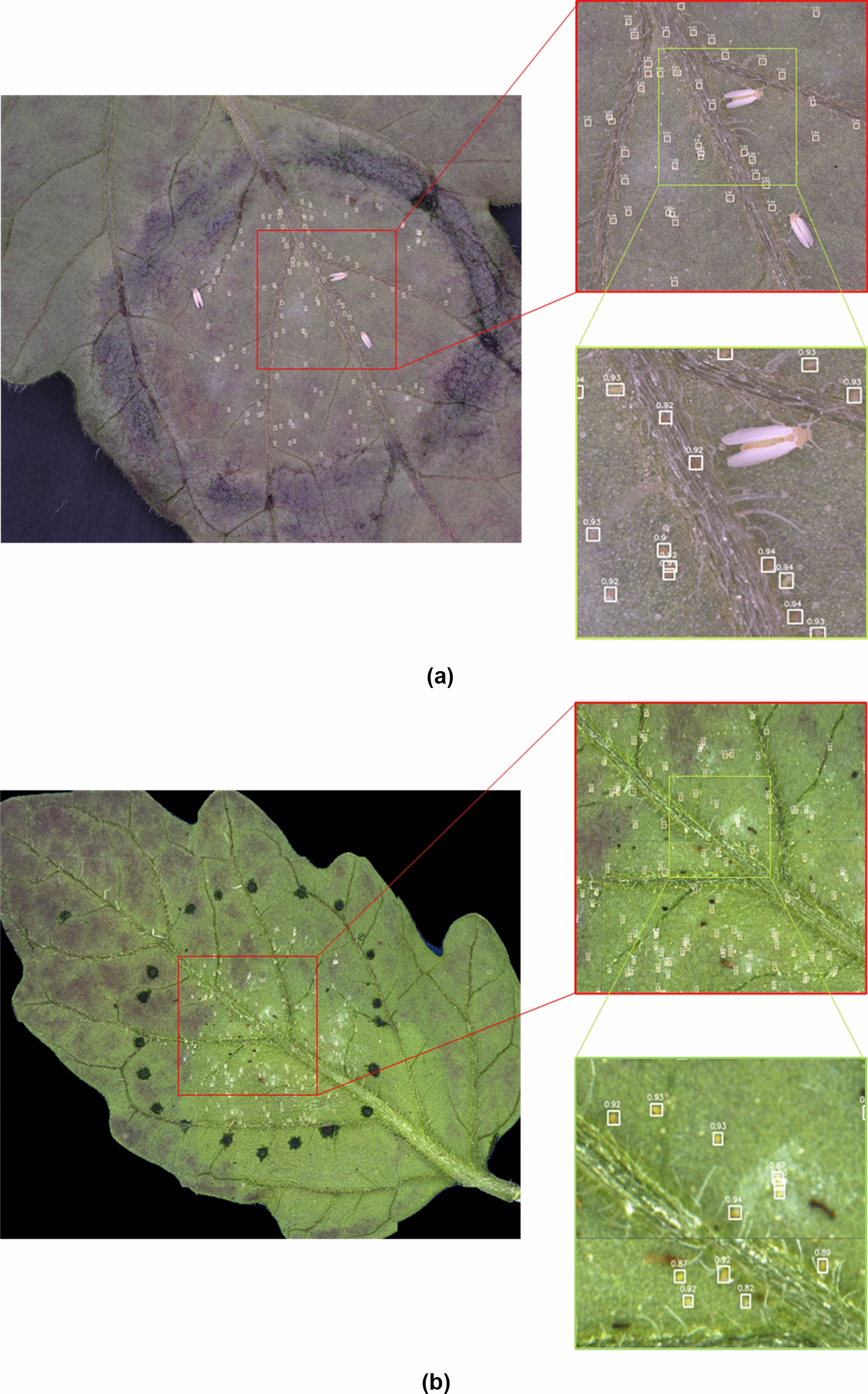
Sample automated egg quantification results using different imaging setups: **a** commercial microscope; and **b** AutoEnto device

The quality of detections was also visually evaluated, as shown in Fig. 5. It can be noticed that most whitefly eggs can be easily detected by the algorithm, with high confidence scores of about 0.9 (Fig. 5a). The algorithm also performed well on the AutoEnto leaf images even though the lighting and white balance was slightly different from the settings of the commercial microscope (Fig. 5b). This indicates that the algorithm is very adaptive to changes in imaging conditions.

**Fig. 6:**
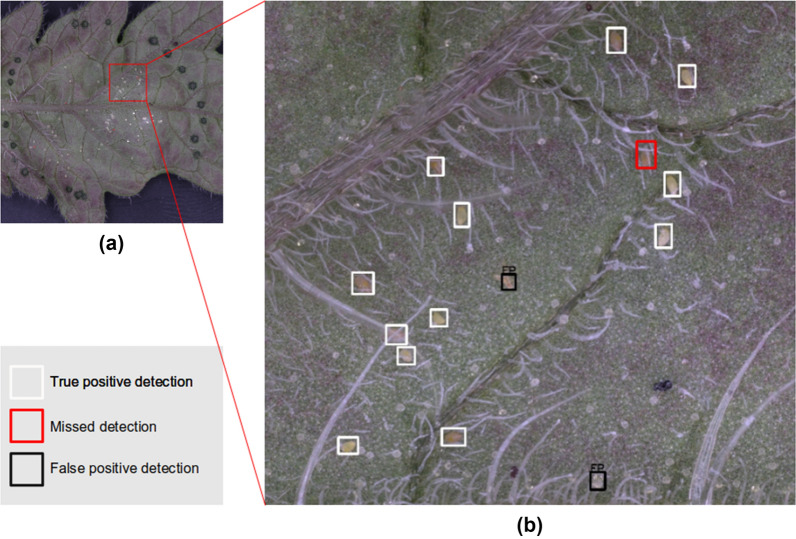
Sample errors obtained by the automated egg quantification algorithm

Some of the errors obtained by the algorithm are shown in Fig. 6. As seen from the upper right of Fig. 6b, a whitefly egg was not successfully detected since it was slightly covered by a strand of trichome. Meanwhile, some plant material was falsely detected as an egg in the middle of Fig. 6b, while a trichome cuticle was also falsely detected at the bottom of Fig. 6b. In the future, these errors can be minimized by collecting more training images, most especially of different egg colors and trichome densities, and possibly devising other post-processing strategies.

### Determination of insect plant resistance and susceptibility

**Fig. 7:**
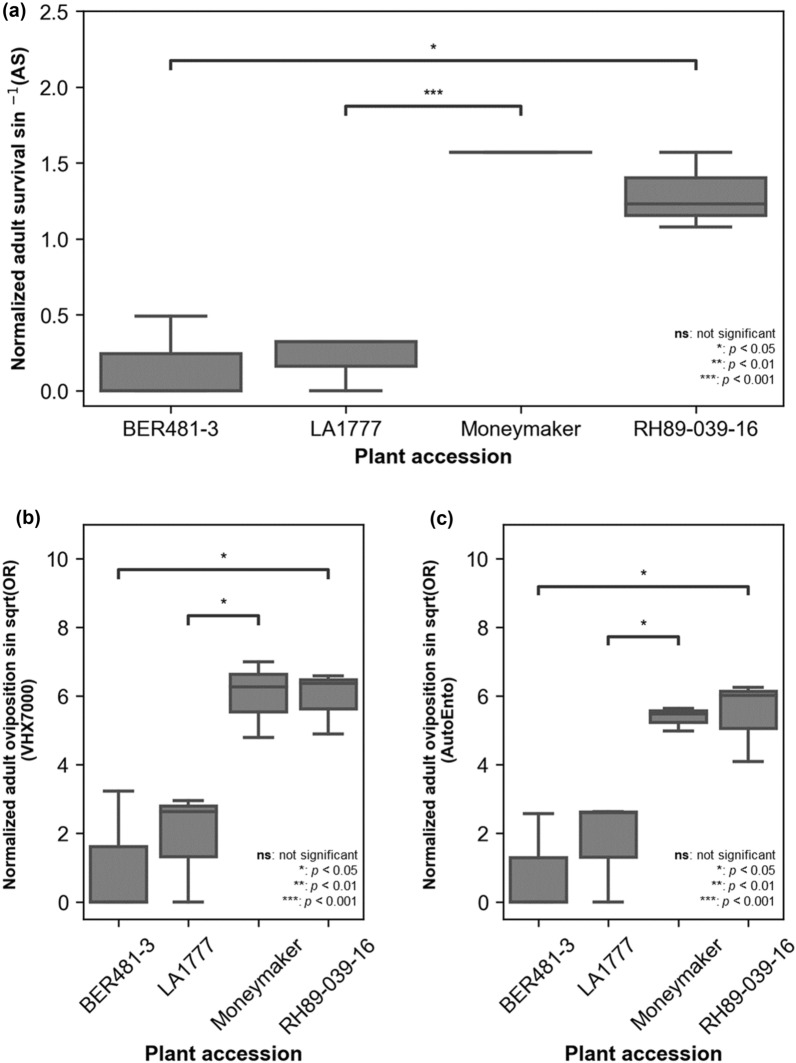
Plant–insect resistance and susceptibility analysis: **a** normalized adult survival rate; **b** normalized oviposition rate measured using the processed VHX7000 images; and **c** normalized oviposition rate measured using the processed AutoEnto images

**Fig. 8:**
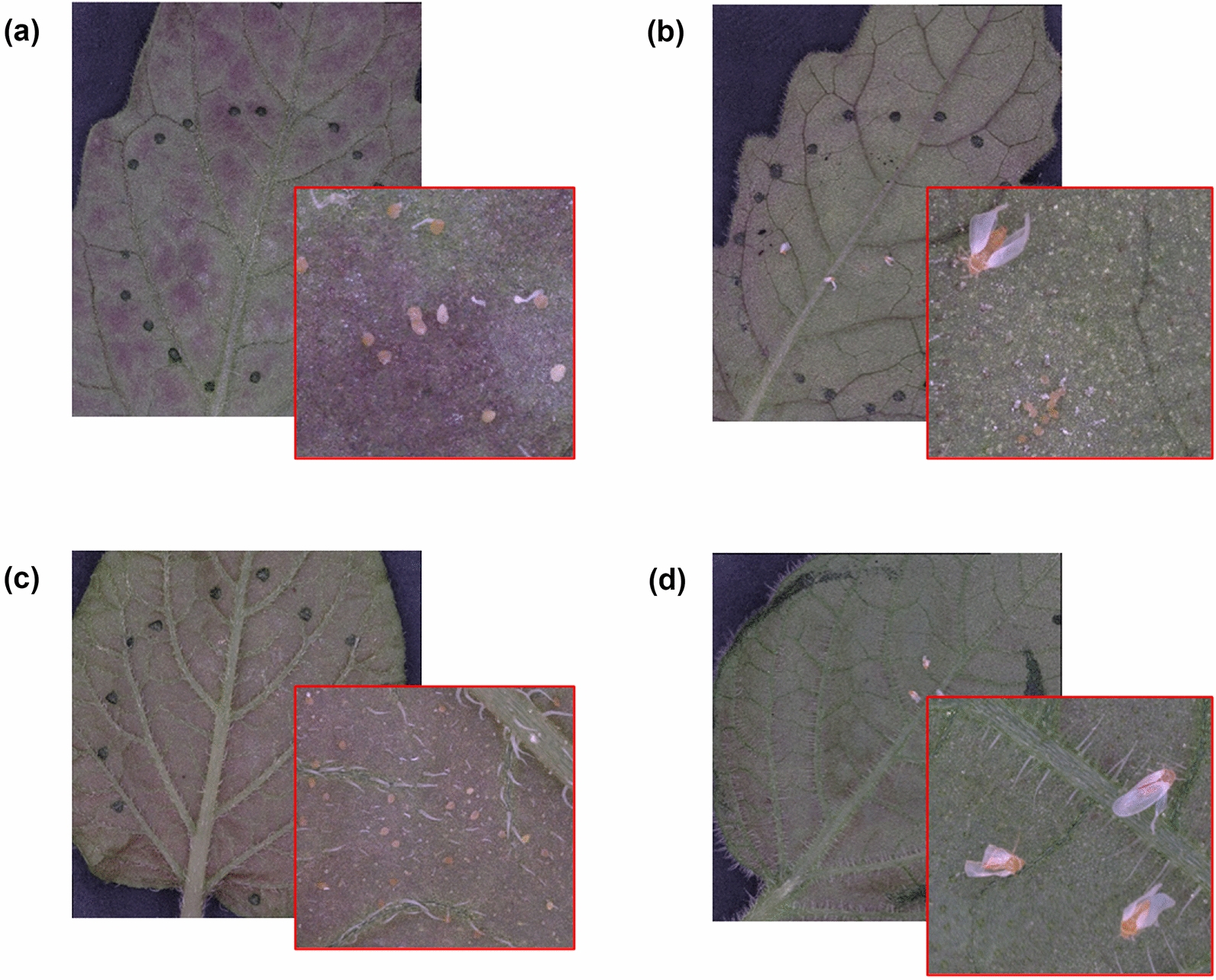
Sample leaf images taken from each accession: **a** *S. lycopersicum* cv. Moneymaker; **b** *S. habrochaites* LA1777; **c** *S. tuberosum* cv. RH89-039-16; and **d** *S. berthaultii* BER481-3, showing oviposition

The plant–insect resistance analysis results are shown in Fig. 7 and Table 2, while sample leaf images of each accession are shown in Fig. 8. In this experiment, BER481-3 (*S. berthaultii*) and LA1777 (*S. habrochaites*) were selected as accessions that are highly resistant to *B. tabaci* and *Trialeurodes vaporariorum* [4, 22], BER481-3 is a newly reported *S. berthaultii* accession that is resistant to whitefly. Meanwhile, the cultivated tomato Moneymaker and potato RH89-039-16 accessions are known susceptible genotypes, not only for insect resistance [7, 40]. Based on statistical analysis, both Moneymaker (*p* < 0.001, *n* = 3) and RH89-039-16 (*p* < 0.05, *n* = 3) showed significantly higher adult survival (Fig. 7a) and oviposition (*p* < 0.05, *n* = 3) (Fig. 7b) compared to the resistant accessions. Generally, Moneymaker and RH89-039-16 had higher oviposition on the abaxial surface of the leaf compared to the wild accessions, as can be observed in Fig. 8a, c, respectively.

**Table 2:** Whitefly assay result for female survival and oviposition

Species	Accession	Clip cage 1				Clip cage 2			
		Alive whiteflies	Dead whiteflies	Whitefly eggs		Alive whiteflies	Dead whiteflies	Whitefly eggs	
				VHX7000	AutoEnto			VHX7000	AutoEnto
*S. habrochaites*	LA1777	0	5	0	0	0	5	0	0
	LA1777	1	4	34	37	0	5	14	0
	LA1777	0	5	38	38	1	4	0	0
*S. lycopersicum*	Moneymaker	5	0	128	171	5	0	102	77
	Moneymaker	3	0	119	91	5	0	195	149
	Moneymaker	4	0	220	59	5	0	220	227
*S. tuberosum*	RH89-039-16	3	1	134	113	5	0	210	194
	RH89-039-16	3	1	140	119	4	1	207	194
	RH89-039-16	5	0	157	108	4	0	58	43
*S. berthaultii*	BER481-3	0	5	52	33	0	5	0	0
	BER481-3	0	5	0	0	0	5	0	0
	BER481-3	0	5	0	0	2	2	0	0

### Imaging device evaluation

One of the goals of this research was to determine which imaging devices are suitable for quantifying whitefly eggs on leaf samples. The results in Fig. 7c show that, despite the number of false positives/negatives of eggs counted due to the differences in leaf surface morphology (e.g., color and trichome composition), the statistical conclusions that can be drawn from both imaging devices were similar. The VHX7000 microscope is the most recent high-end version of electronic stereomicroscope from the VHX Keyence line. Up to date, researchers use this line of microscope to capture fast high-resolution z-stacked stitched images from plant cells to insects [31, 32]. In this research, the acquisition of each leaf sample using the VHX7000 takes about 2 min. On the other hand, AutoEnto costs approximately €5000 and it can take an image of a leaf sample in a dish in about 2 min, but it can be programmed to acquire 9 dishes at a time. Additionally, the accompanying computer of the AutoEnto may be programmed to upload images and record whitefly egg counts automatically. In this research, it was found that image quality was a disadvantage of the AutoEnto, but it can be used for faster image acquisition. Nevertheless, AutoEnto serves as a budget-friendlier customized device alternative but VHX7000 can acquire sharper images.

### Web application deployment

**Fig. 9:**
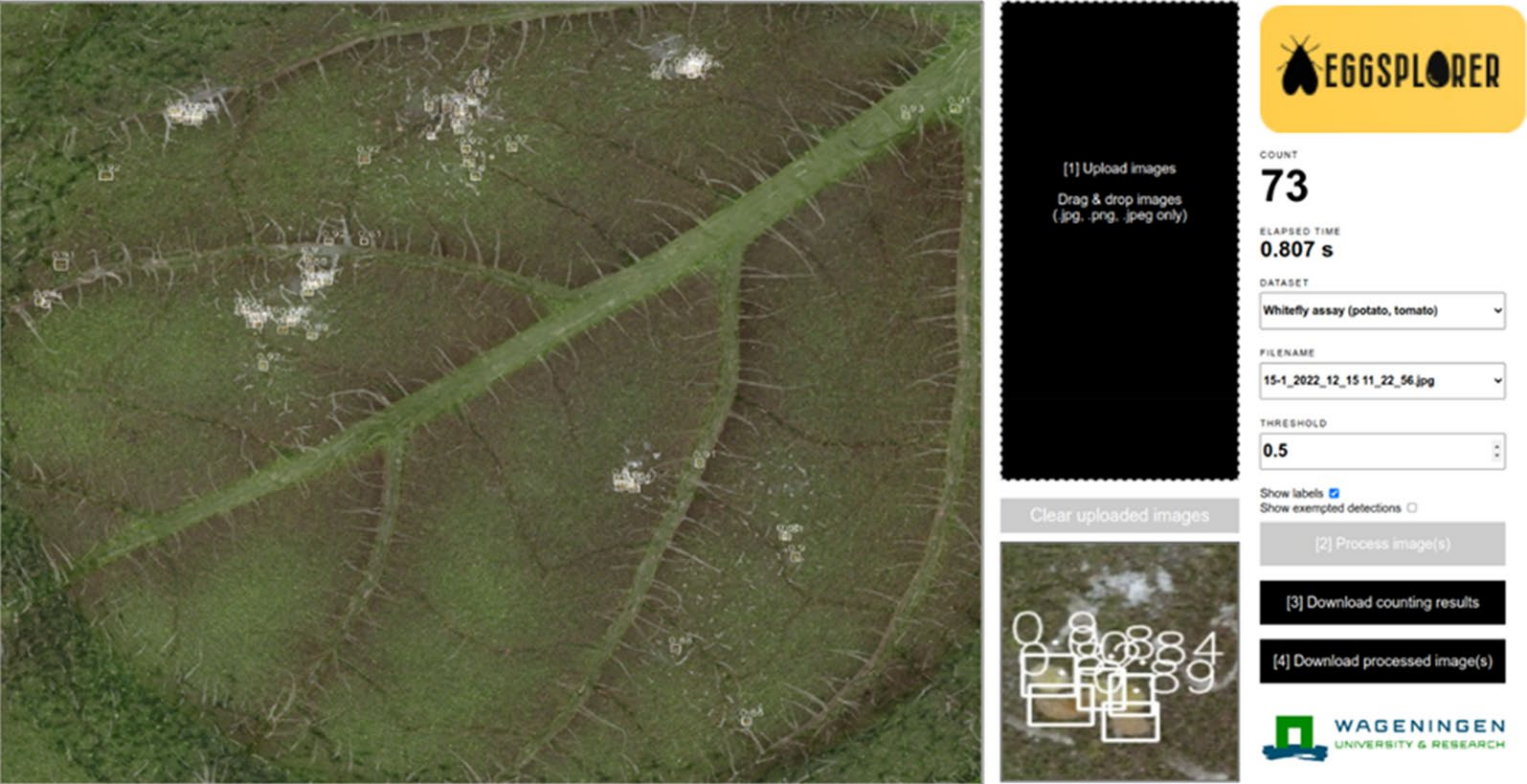
Screenshot of the Eggsplorer web application

The automated egg quantification algorithm was deployed in a web application that we named Eggsplorer. Eggsplorer was written using Python, JavaScript, and HTML programming languages, with the support of Flask micro web framework. A screenshot of the web application is shown in Fig. 9. As shown, the user can drag and drop images to the web application for uploading. The user can also configure the classification confidence threshold if unwanted detections are to be ignored. Once all leaf images are uploaded, each leaf image is processed on the server. The detection results are shown in the web application. Finally, the counting results can be downloaded as a.xlsx file while the processed images can be downloaded as a.zip file. Currently, the web application can only be accessed in the WUR intranet but may also be opened to interested researchers.

## Conclusion

A novel method for determining plant–insect resistance and susceptibility, with the assistance of an automated egg quantification tool, is presented in this work. The results show that the automated quantification tool could count whitefly eggs obtained from two different imaging setups. Algorithm testing results showed that the quantification tool can be used on images generated from various microscopes. Users of other microscopes can simply upload their own images in the web application and quantify the whitefly eggs found in their leaf samples. Alternatively, a custom-built imaging setup, such as the AutoEnto, can also be used for faster image acquisition and sampling.

The procedures presented herein can be a reference to other researchers for determining plant–insect resistance and susceptibility in a quantitative and practical manner. In the future, images from other leaves may also be obtained to train new models and make the web-based application more versatile. This can be done by incorporating images of other insect eggs such as mites, thrips, and other harmful insect pests, to build a universal platform for determining plant–insect resistance.

## Data Availability

The datasets used and analyzed during the current study are available from the corresponding author on reasonable request.

## Abbreviations

AC: Automatic counts
AS: Adult survival
IoU: Intersection-over-Union
OR: Oviposition rate
TC: True counts
TP: True positive
